# Integrated Network Pharmacology and Metabonomics to Reveal the Myocardial Protection Effect of Huang-Lian-Jie-Du-Tang on Myocardial Ischemia

**DOI:** 10.3389/fphar.2020.589175

**Published:** 2021-02-04

**Authors:** Li Li, Weixing Dai, Wenting Li, Yumao Zhang, Yanqin Wu, Chenfeng Guan, Anye Zhang, Hui Huang, Yuzhen Li

**Affiliations:** ^1^ Department of Pharmacy, The Eighth Affiliated Hospital, Sun Yat-sen University, Shenzhen, China; ^2^ Department of Chemical and Biological Engineering, Hong Kong University of Science and Technology, Kowloon, China; ^3^ Department of Gastroenterology, The Eighth Affiliated Hospital, Sun Yat-sen University, Shenzhen, China; ^4^ Department of Cardiovascular, The Eighth Affiliated Hospital, Sun Yat-sen University, Shenzhen, China

**Keywords:** HLJDT, myocardial ischemia, network pharmacology, metabonomics, multitarget

## Abstract

Myocardial ischemia (MI) is one of the most common cardiovascular diseases with high incidence and mortality. Huang-Lian-Jie-Du-Tang (HLJDT) is a classic traditional Chinese prescription to clear “heat” and “poison”. In this study, we used a deliberate strategy integrating the methods of network pharmacology, pharmacodynamics, and metabonomics to investigate the molecular mechanism and potential targets of HLJDT in the treatment of MI. Firstly, by a network pharmacology approach, a global view of the potential compound-target-pathway network based on network pharmacology was constructed to provide a preliminary understanding of bioactive compounds and related targets of HLJDT for elucidating its molecular mechanisms in MI. Subsequently, *in vivo* efficacy of HLJDT was validated in a rat model. Meanwhile, the corresponding metabonomic profiles were used to explore differentially induced metabolic markers thus providing the metabolic mechanism of HLJDT in treating MI. The results demonstrated the myocardial protection effect of HLJDT on ischemia by a multicomponent-multitarget mode. This study highlights the reliability and effectiveness of a network pharmacology-based approach that identifies and validates the complex of natural compounds in HLJDT for illustrating the mechanism for the treatment of MI.

## Introduction

Acute myocardial ischemia (AMI) refers to a pathological state in which blood perfusion is reduced due to coronary artery occlusion, insufficient blood flow, or oxygen supply. It is one of the most common cardiovascular diseases with a high incidence rate and high mortality rate (Turer and Hill, 2010; Davidson et al., 2019). Studies have shown that the pathogenesis of AMI is closely related to biological pathways including energy metabolism, oxidative stress (Kaul and Ito, 2004; Luqman et al., 2007), apoptosis (Garg et al., 2003), calcium homeostasis (Katopodis et al., 1997), angiogenesis (Lee et al., 2000), and inflammatory and immune responses (Rui et al., 2005; Arslan et al., 2011). In the theory of traditional Chinese medicine (TCM), AMI belongs to the category of “chest obstruction” and “cardialgia”, which can be treated by “invigorating the circulation of Qi and blood”, “dispersing stagnation”, and “dredging collaterals”. Various Chinese herbal medicines, such as Salvia miltiorrhiza, Rhodiolarosea, Forsythia suspensa, Sini Tang, and Tongxinluo, have been reported to improve myocardial ischemia and protect the heart (Liu et al., 2013; Guo et al., 2016), which indicates the advantage of TCM in myocardial protection. Nevertheless, as the multi-component, multi-target, and multi-channel synergistic characteristics of TCM, it is inappropriate to use the mode of “one drug-one target” to elaborate its mechanism of action. Moreover, it is difficult to carry out systematic research on the level of tissues, organs, cells, and molecules, which remain an obstacle for the modernization of TCM (Wang et al., 2012).

Network pharmacology is one of the emerging strategies based on multi-disciplinary technologies such as system biology, multi-directional pharmacology, computational biology, and network analysis, which systematically reveals the core molecular targets and pharmacodynamics methods of TCM (Hopkins, 2008; Li and Zhang, 2013). In this approach, the multi-level relationship between “drug-compound-target-pathway-disease” is established by using various technologies including Omics technology, high-throughput screening, network visualization, or network analysis. It helps us understand the molecular basis of disease, predicts the pharmacological mechanism, and finds herbal compounds of high efficiency and low toxicity.

Huang-Lian-Jie-Du-Tang (HLJDT) is a classic traditional Chinese prescription to clear “heat” and “poison”, which was first published in Gehong’s “elbow reserve emergency prescription”. It consists of the rhizoma of *Coptis chinensis* Franch (Huanglian 9 g), the radix of *Scutellaria baicalensis* Georgi (Huangqin 6 g), the cortex of *Phellodendron chinense* C.K. Schneid (Huangbo 6 g) and the fructus of *Gardenia jasminoides* J. Ellis (Zhizi, 9 g) (Shen et al., 2003). Modern pharmacology research shows that HLJDT has obvious anti-inflammatory, antibacterial, anti-endotoxin, and antipyretic effects (Li et al., 2012; Lv et al., 2017). Recent studies showed that HLJDT could significantly reduce cerebral ischemia injury, cholesterol, etc. (Sekiya et al., 2005; Wang et al., 2013) Studies showed that the effective components in HLJDT could mainly be divided into three categories: 1) alkaloids: mainly from the rhizoma of *Coptis chinensis* Franch and the cortex of *Phellodendron chinense* C.K. Schneid. Berberine and Phellodendron amurense are the focuses of current studies. 2) Flavonoids: mainly from the radix of *Scutellaria baicalensis* Georgi. The representative components are baicalein, scutellarin, and baicalin. 3) Iridoids: mainly from the fructus of *Gardenia jasminoides* J. Ellis and its representative component is gardenside (Han et al., 2007; Ma et al., 2009; Chen et al., 2010). Although there have been many studies on the efficacy and mechanism of HLJDT, most of them focus on a single component and the multi-component and multi-target mechanism is still unclear.

**FIGURE 1:**
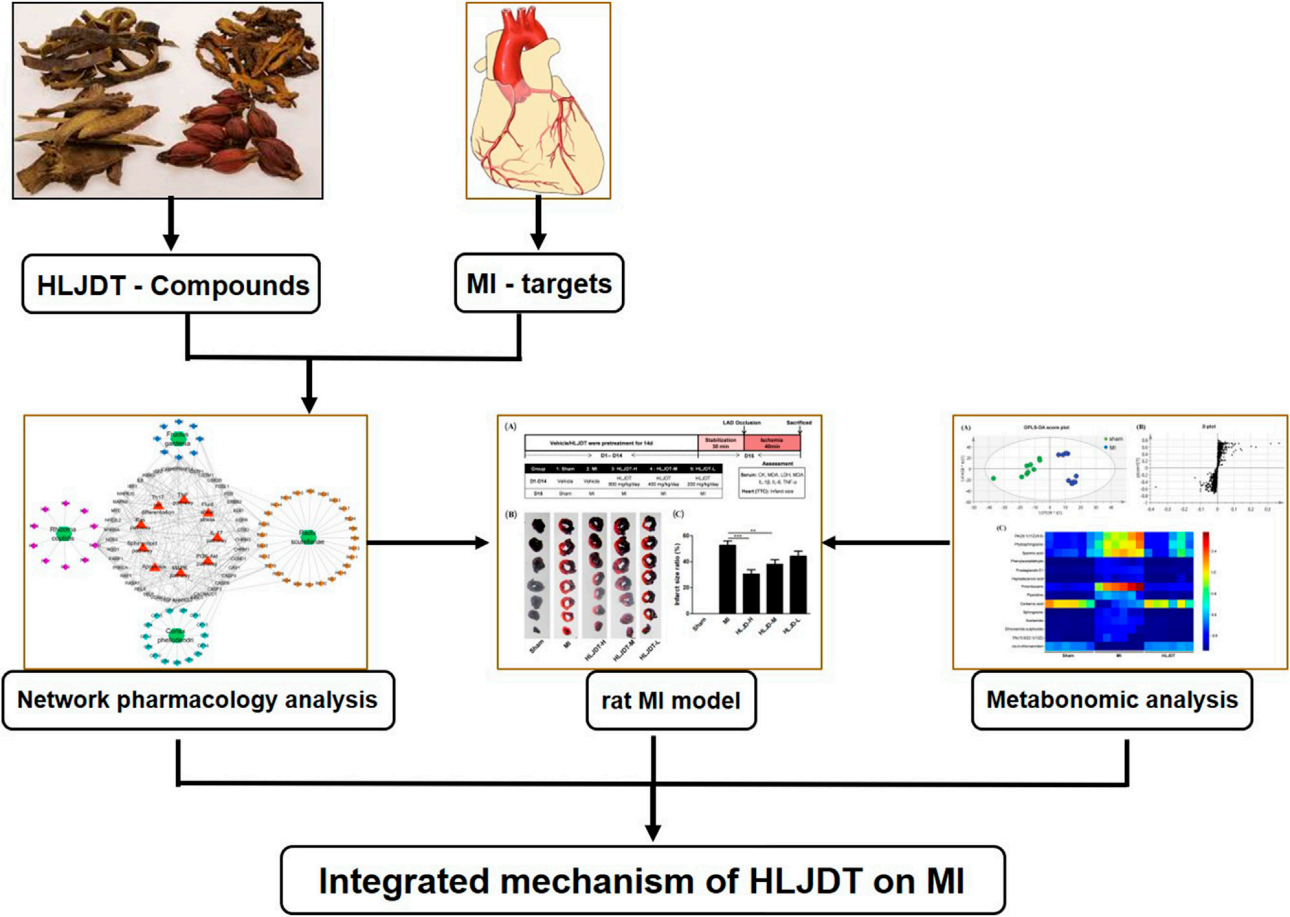
Integrated flowchart for elucidating the mechanism of HLJDT in the treatment of MI. Firstly, a global view of the potential compound–target–pathway network based on network pharmacology was constructed to provide a preliminary mechanisms prediction the of HLJDT for elucidating its molecular mechanisms in MI. Subsequently, in vivo efficacy of HLJDT was validated in a rat model. Meanwhile, the corresponding metabonomic profiles were used to explore differentially induced metabolic markers thus providing the metabolic mechanism of HLJDT in treating MI. These results will demonstrate the myocardial protection effect and illustrating the mechanism of HLJDT for the treatment of MI.

In this study, we used a deliberate strategy integrating the methods of network pharmacology, pharmacodynamics, and metabonomics to investigate the molecular mechanism and potential target of HLJDT for treatment of MI. Firstly, an MI-related “potential compounds-target-pathway” regulatory network was generated by network pharmacology to identify potential bioactive compounds and related targets of HLJDT. Subsequently, *in vivo* experimental verification of the efficacy was performed in a rat MI model by ligation of the coronary artery. The metabonomics approach was applied to explore differentially induced metabolic markers, thus providing the metabolic mechanism of HLJDT in treating MI. This study highlights the reliability and effectiveness of a network pharmacology-based approach that identifies and validates the complexity of natural compounds in HLJDT for illustrating the mechanism of action in the treatment of MI. The overall flowchart for elucidating the mechanism of HLJDT in the treatment of MI is illustrated in Figure 1.

## Materials and Methods

### Network Pharmacology Analysis

#### Predicting Potential Gene Targets of Chemical Composition in Huang-Lian-Jie-Du-Tang

The chemical compositions of HLJDT were collected from the Traditional Chinese Medicine Systems Pharmacology database (TCMSP, http://lsp.nwu.edu.cn/) (Ru et al., 2014), which is a unique database platform of Chinese herbal medicine system pharmacology for capturing the relationship among drugs, targets, and diseases. Those meeting the criteria of bioavailability (OB) ≥30% and drug-likeness (DL) ≥0.1 (Xu et al., 2012) were used for further analysis. The HPLC chromatogram of HLJDT obtained from the methanol extract of HLJDT is shown in Supplementary Figure S1. The potential gene targets of the major compounds in HLJDT were searched from the TCMSP and PubMed databases. Totally, 448 HLJDT-related targets were obtained (Supplementary Table S2).

#### MI-Associated Target Genes

The target gene number (PDB ID number) was converted into a gene name corresponding to the Genecards database through the UniProt database. The key words “myocardial ischemia” or “myocardial infarction” were input into the Genecards database (http://www.genecards.org, a database providing a detailed genome, proteome, transcription, genetic, and functional overview of all known and predicted human genes) and the Online Mendelian Inheritance in Man database (OMIM) to collect target genes related to MI. The common target genes were screened out as the target of HLJDT in the treatment of MI.

#### Pathway Enrichment and Construction of the C-T-P Network

In order to explain the potential role of the active components in HLJDT in gene function and signaling pathways, we used DAVID (database for Annotation, Visualization and Integrated Discovery) to analyze the KEGG pathways of the predicted targets. The results of KEGG enrichment analysis were visualized using the Omicshare online analysis platform. The components-targets-pathway (C-P-T) network of HLJDT for treating MI was constructed by utilizing the network-visualization software Cytoscapev3.6.1.

### Experimental Validation of Huang-Lian-Jie-Du-Tang on MI *in vivo*

#### Animals

Male Sprague–Dawley (SD) rats (180–200 g) were purchased from the SLAC Laboratory Animal Co., Ltd. (Shanghai, China). The animals were housed in standard pathogen-free cages in a climate-controlled environment with a 12 h light/12 h dark cycle. Sterile food and water were naturally provided. All procedures were conducted in accordance with the guidelines for care and use of laboratory animals from the Second Military Medical University and government.

#### Animals Grouping and Drug Administration

**FIGURE 2:**
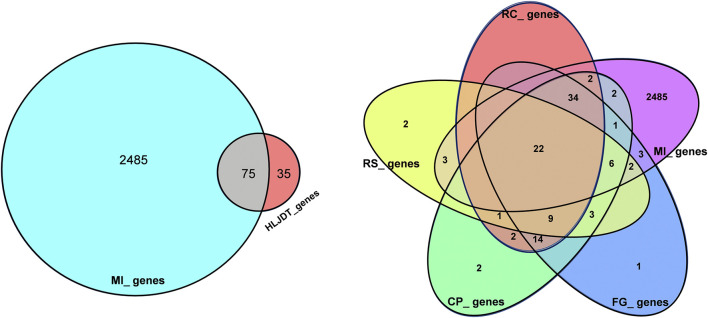
Overlaps between different gene sets. **(A)** Overlaps between MI diseases genes from HLJDT-related genes; **(B)** Overlaps between different gene sets from MI and 4 main herbs in HLJDT prescription. RC*‐Rhizoma coptidis, RS-Radix scutellariae, CP‐Cortex phellodendri, FG-Fructus gardenia*.

The experimental procedure of the *in vivo* MI rat model is shown in Figure 2A. Fifty SD rats were randomly divided into five groups (10 rats per group): 1) a sham group (vehicle-treated + sham); 2) an MI group (vehicle-treated + MI); 3) a high-dose HLJDT group (800 mg/kg/day); 4) a middle-dose HLJDT group (400 mg/kg/day); and 5) a low-dose HLJDT group (200 mg/kg/day). The vehicle and HLJDT were pretreated by gavage once per day for 14 consecutive days before the establishment of the MI model.

#### Establishment of the Rat MI Model

The 50 experimental rats were anesthetized with isoflurane inhalation and needle electrodes were inserted into the subcutaneous limbs. A catheter containing 0.1% heparin saline was inserted into the left ventricle through the left common carotid artery. Electrocardiograph (ECG) and left ventricular hemodynamic changes were recorded by a BL-410 biological function experiment system and the changes of the S-T segment were used to determine the success of modeling. Endotracheal intubation and artificial ventilators were used for mechanical ventilation. The MI model was established by ligating the left anterior descending coronary artery (LADCA) (Wang et al., 2002). After the LAD was exposed between the left auricle and the pulmonary conus, a small section of polyethylene tube was put through with a 4–0 black silk ligature to block LAD blood flow for 30 min. The sham group was handled using the same operation process without the ligation step. Five rats died throughout the experiment and the other 45 rats who survived were successfully modeled (the modeling rate was up to 90%), including 10 rats in the group (1), eight rats in group (2), 10 rats in group (3), eight rats in group (4), and nine rats in group (5).

#### Sample Collection and Detection

The experimental rats were sacrificed 30 min after modeling. Immediately, blood was collected from the abdominal aorta. The supernatant serum obtained was collected and stored at −20°C for analysis of the biochemical indexes including CK, MDA, SOD, LDH and IL-1β, IL-6, IL-17, and TNF-α. The remaining serum was stored at −80°C for metabonomic analysis. Simultaneously, the hearts were rapidly removed for 2,3,5-triphenyltetrazolium chloride (TTC) immersion to determine the myocardial infarct size.

### Metabonomic Analysis

#### UPLC-Q-TOF/MS Measurement and Data Analysis

Metabonomic analysis was performed by the Agilent 1,290 Infinity Ⅱ UHPLC system coupled to an Agilent 6545 UHD and Accurate-Mass Q-TOF/MS. Mobile phase: A: aqueous solution. B: acetonitrile solution. Flow rate: 0.35 ml/min. Column temperature: 25°C. Injection volume: 2 μL. Gradient elution condition optimized: 0–2 min, 1% B; 2–7 min, 1–15% B; 7–9 min, 15–50% B; 9–13 min, 50–95% B; 13–15 min, 95% B. Post time was set as 5 min. Mass spectrometry was operated in both positive and negative ion modes. Differential metabolites were further identified by MS/MS with collision energy of 10 v, 20 v, and 40 v. Raw data were converted by the self-contained software of the Agilent system. Then the peak was identified by the XCMS program in the R software platform. The peel data were subjected to internal standard normalization and weight normalization and the flesh data were subjected to internal standard normalization. Visualization matrices containing sample name, m/z-RT pair, and peak area were obtained. For the peel simples, 1,428 features were obtained in the positive mode. After editing, the data matrices were imported into SIMCA-P 13.0 After preprocessing, the data were analyzed by Orthogonal Partial Least Square Discriminate Analysis (OPLS-DA).

#### Qualitative Analysis of Metabolites and Screening of Differential Metabolites

The metabolites were qualitatively analyzed by searching the self-built standard substance database according to the results of the secondary spectrum and then the METLIN, HMDB, HMDB-SERUM, KEGG, CHEBI, LIPID, and other public databases were searched to provide alternative substances. In our study, the Variable Importance in Projection (VIP, threshold >1) of the first principal component of the OPLS-DA model and the *p* value (threshold <0.05) of Student’s t-test were used to find differentially expressed metabolites.

### Statistical Analysis

Data were analyzed by one-way analysis of variance (ANOVA) followed by a Dunnett's test or a Kruskal-Wallis ANOVA on Ranks followed by a Dunn’s test for multiple comparisons and expressed as the means ± SEM. *p < 0.05 was considered a significant difference.

## Results

### Prediction of the Pharmacological Mechanism of Huang-Lian-Jie-Du-Tang on MI by a Network Pharmacology Approach

#### Herbal Compounds in Huang-Lian-Jie-Du-Tang

Using the TCMSP database, 429 compounds were retrieved, where 48 were in *Rhizoma coptidis*, 143 were in *Radix scutellariae*, 140 were in *Cortex phellodendri*, and 98 were in *Fructus gardenia*. With the criteria of OB larger than 30% and DL larger than 0.18, 106 chemical ingredients were screened out, where 14 were in *Rhizoma coptidis*, 36 were in *Radix scutellariae*, 37 were in *Cortex phellodendri*, and 15 were in *Fructus gardenia*. After taking out the duplicated parts, 84 chemical constituents were accepted, and their structures were retrieved from the PubChem database (Supplementary Table S1). Finally, 59 chemical ingredients were screened out for further target prediction analysis (Table 1).

**TABLE 1:** Compounds in HLJDT with oral bioavailability (OB) larger than 30% and drug-likeness (DL) larger than 0.18.

Comp	Herbal compound	OB%	DL	Herb	Comp	Herbal compound	OB%	DL	Herb
1	Fumarine	59.26	0.83	CP1	31	Berberine	36.86	0.78	RC6
2	Cavidine	35.64	0.81	CP2	32	(R)-canadine	55.37	0.77	RC7
3	Chelerythrine	34.18	0.78	CP3	33	Berberrubine	35.74	0.73	RC8
4	(S)-canadine	53.83	0.77	CP4	34	Palmatine	64.6	0.65	RC9
5	Delta 7-stigmastenol	37.42	0.75	CP5	35	Sitosterol	36.91	0.75	RS1
6	Poriferast-5-en-3beta-ol	36.91	0.75	CP6	36	5,7,2,5-Tetrahydroxy-8,6-dimethoxyflavone	33.82	0.45	RS2
7	Beta-sitosterol	36.91	0.75	CP7	37	NEOBAICALEIN	104.34	0.44	RS3
8	Thalifendine	44.41	0.73	CP8	38	Skullcapflavone II	69.51	0.44	RS4
9	Campesterol	37.58	0.71	CP9	39	Diop	43.59	0.39	RS5
10	Rutaecarpine	40.3	0.6	CP10	40	Rivularin	37.94	0.37	RS6
11	Isocorypalmine	35.77	0.59	CP11	41	5,2′-dihydroxy-6,7,8-trimethoxyflavone	31.71	0.35	RS7
12	Phellavin_qt	35.86	0.44	CP12	42	Salvigenin	49.07	0.33	RS8
13	Dehydrotanshinone II A	43.76	0.4	CP13	43	5,2′,6′-trihydroxy-7,8-dimethoxyflavone	45.05	0.33	RS9
14	phellamurin_qt	56.6	0.39	CP14	44	Pangolin	76.26	0.29	RS10
15	Phellopterin	40.19	0.28	CP15	45	5,7,4′-trihydroxy-6-methoxyflavanone	36.63	0.27	RS11
16	Stigmasterol	43.83	0.76	FG1	46	5,7,4′-trihydroxy-8-methoxyflavone	36.56	0.27	RS12
17	Sudan III	84.07	0.59	FG2	47	5,7,4′-trihydroxy-8-methoxyflavanone	74.24	0.26	RS13
18	5-Hydroxy-7-methoxy-2-(3,4,5-trimethoxyphenyl)chromone	51.96	0.41	FG3	48	Moslosooflavone	44.09	0.25	RS14
19	3-Methylkempferol	60.16	0.26	FG4	49	Ent-epicatechin	48.96	0.24	RS15
20	Crocetin	35.3	0.26	FG5	50	Eriodyctiol (flavanone)	41.35	0.24	RS16
21	Kaempferol	41.88	0.24	FG6	51	Carthamidin	41.15	0.24	RS17
22	Ammidin	34.55	0.22	FG7	52	5,7,2′,6′-tetrahydroxyflavone	37.01	0.24	RS18
23	Mandenol	42	0.19	FG8	53	Acacetin	34.97	0.24	RS19
24	Worenine	45.83	0.87	RC1	54	DIHYDROOROXYLIN	66.06	0.23	RS20
25	Quercetin	46.43	0.28	RC10	55	Oroxylin a	41.37	0.23	RS21
26	Coptisine	30.67	0.86	RC2	56	Wogonin	30.68	0.23	RS22
27	Berlambine	36.68	0.82	RC3	57	Dihydrobaicalin_qt	40.04	0.21	RS23
28	CorchorosideA_qt	104.95	0.78	RC4	58	Norwogonin	39.4	0.21	RS24
29	Epiberberine	43.09	0.78	RC5	59	Baicalein	33.52	0.21	RS25
30	Fumarine	59.26	0.83	CP1					

#### Identification of Huang-Lian-Jie-Du-Tang-Related Targets

**FIGURE 3:**
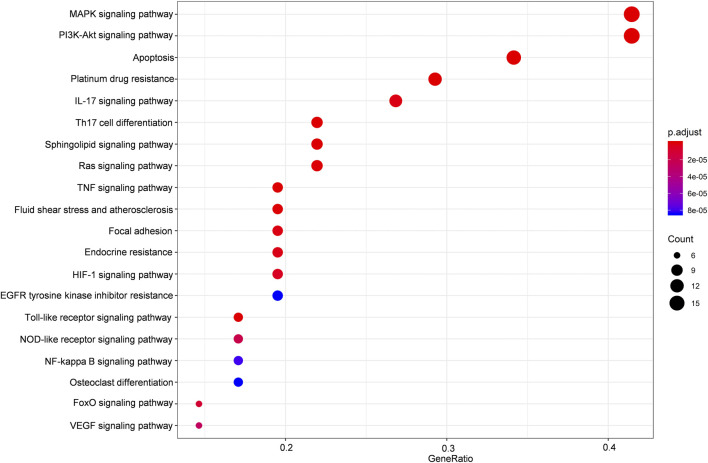
KEGG enrichment analysis of the predicted targets of HLJDT for MI treatment. Dot plot showing the top 20 KEGG pathways: the size of the dots corresponds to the number of genes annotated in the entry, and the color of the dots corresponds to the corrected P‐value.

Overall, 2,138 known distinct MI-related targets were eventually collected from the GenesCard database (Supplementary Table S3) and 375 were collected from the OMIM database (Supplementary Table S4), which included almost all the target genes related to MI that have been identified or are being studied. The shared potential target genes of HLJDT with MI-related target genes that correspond to disease progression and treatment were considered potential targets of HLJDT. Among the 2485 MI-related target genes, 75 genes were closely related to HLJDT (Table 2). Figure 3 shows the number of shared overlap targets by HLJDT and MI-related targets.

**TABLE 2:** Myocardial ischemia (MI) related genes targeted by active compounds of HLJDT.

No.	GC id	Gene symbol	Description	Gifts	Relevance score
1	GC06P043770	VEGFA	Vascular endothelial growth factor A	56	42.46
2	GC07M095297	PON1	Paraoxonase 1	53	34.38
3	GC01M159682	CRP	C-reactive protein	53	30.85
4	GC07P022765	IL6	Interleukin 6	55	29.21
5	GC07P150990	NOS3	Nitric oxide synthase 3	57	28.06
6	GC13P113105	F7	Coagulation factor VII	53	25.95
7	GC06P151656	ESR1	Estrogen receptor 1	60	22.36
8	GC04M184627	CASP3	Caspase 3	57	21.69
9	GC19P010270	ICAM1	Intercellular adhesion molecule 1	57	21.05
10	GC14P061695	HIF1A	Hypoxia inducible factor 1 subunit alpha	54	20.97
11	GC01M169722	SELE	Selectin E	50	18.15
12	GC03P012328	PPARG	Peroxisome proliferator activated receptor γ	59	15
13	GC01P100719	VCAM1	Vascular cell adhesion molecule 1	52	14.66
14	GC01M226360	PARP1	Poly (ADP-ribose) polymerase 1	56	13.37
15	GC18M063123	BCL2	BCL2 apoptosis regulator	59	13.22
16	GC09P122370	PTGS1	Prostaglandin-endoperoxide synthase 1	53	12.81
17	GC04M148078	NR3C2	Nuclear receptor subfamily 3 group C2	54	11.91
18	GC10P094938	CYP2C9	Cytochrome P450 family 2 subfamily C9	56	11.21
19	GC01M015565	CASP9	Caspase 9	54	8.69
20	GC07P116524	CAV1	Caveolin 1	54	8.02
21	GC07M134442	AKR1B1	Aldo-keto reductase family 1 member B	54	6.9
22	GC10P045338	ALOX5	Arachidonate 5-lipoxygenase	54	6.8
23	GC14P075278	FOS	Fos proto-oncogene	57	6.58
24	GC10P048306	MAPK8	Mitogen-activated protein kinase 8	56	6.33
25	GC07M045912	IGFBP3	Insulin like growth factor binding protein 3	51	6.13
26	GC02M177227	NFE2L2	Nuclear factor, erythroid 2 like 2	54	5.98
27	GC07P076302	HSPB1	Heat shock protein family B (small) member 1	57	5.76
28	GC07P144396	MGAM	Maltase-glucoamylase	48	5.03
29	GC11M100943	PGR	Progesterone receptor	57	5.02
30	GC02P188974	COL3A1	Collagen type III alpha 1 chain	53	4.9
31	GC03M012583	RAF1	Raf-1 proto-oncogene	62	4.42
32	GC17P066302	PRKCA	Protein kinase C alpha	57	4.28
33	GC10P073909	PLAU	Plasminogen activator, urokinase	58	3.96
34	GC05M132481	IRF1	Interferon regulatory factor 1	54	3.8
35	GC03M119821	GSK3B	Glycogen synthase kinase 3 beta	57	3.78
36	GC11M002130	IGF2	Insulin like growth factor 2	54	3.72
37	GC11M063003	CHRM1	Cholinergic receptor muscarinic 1	52	3.64
38	GC02P201233	CASP8	Caspase 8	59	3.57
39	GC04M085990	MAPK10	Mitogen-activated protein kinase 10	57	3.56
40	GC11M001752	CTSD	Cathepsin D	59	3.2
41	GC07P136868	CHRM2	Cholinergic receptor muscarinic 2	54	3.14
42	GC11P067583	GSTP1	Glutathione S-Transferase pi 1	57	3.13
43	GC11M065671	RELA	RELA proto-oncogene, NF-KB subunit	56	3.07
44	GC05M143241	NR3C1	Nuclear receptor subfamily 3 group C1	57	2.76
45	GC11P113974	HTR3A	5-Hydroxytryptamine receptor 3A	53	2.65
46	GC07M099759	CYP3A4	Cytochrome P450 family 3 subfamily A4	56	2.6
47	GC17M037084	ACACA	Acetyl-CoA carboxylase alpha	55	2.53
48	GC17P007282	SLC2A4	Solute carrier family 2 member 4	53	2.51
49	GC01P109687	GSTM1	Glutathione S-Transferase mu 1	48	2.42
50	GC07M100889	ACHE	Acetylcholinesterase (cartwright blood group)	52	2.34
51	GC08M026747	ADRA1A	Adrenoceptor alpha 1A	53	2.31
52	GC08P127735	MYC	MYC proto-oncogene	59	2.23
53	GC04P003804	ADRA2C	Adrenoceptor alpha 2C	51	2.17
54	GC17P078214	BIRC5	Baculoviral IAP repeat containing 5	53	2.16
55	GC0XP067544	AR	Androgen receptor	59	2.11
56	GC11P069641	CCND1	Cyclin D1	59	2.01
57	GC14M035401	NFKBIA	NFKB inhibitor alpha	57	1.98
58	GC14M064084	ESR2	Estrogen receptor 2	56	1.9
59	GC07P055019	EGFR	Epidermal growth factor receptor	62	1.82
60	GC05P087267	RASA1	RAS P21 protein activator 1	53	1.66
61	GC17P039687	ERBB2	Erb-B2 receptor tyrosine kinase 2	62	1.64
62	GC02M038034	CYP1B1	Cytochrome P450 family 1 subfamily B1	56	1.63
63	GC16M069706	NQO1	NAD(P)H quinone dehydrogenase 1	56	1.5
64	GC08P042247	IKBKB	Inhibitor of nuclear factor kappa B kinaseβ	59	1.42
65	GC01M070852	PTGER3	Prostaglandin E receptor 3	53	1.42
66	GC01P239386	CHRM3	Cholinergic receptor muscarinic 3	55	1.32
67	GC02P074796	HK2	Hexokinase 2	53	1.25
68	GC08P144291	HSF1	Heat shock transcription factor 1	51	1.25
69	GC11M065909	FOSL1	FOS like 1, AP-1 transcription factor subunit	50	1.2
70	GC15M074719	CYP1A1	Cytochrome P450 family 1 subfamily A1	53	0.78
71	GC17M082078	FASN	Fatty acid synthase	56	0.73
72	GC07P016916	AHR	Aryl hydrocarbon receptor	56	0.64
73	GC19P040991	CYP2B6	Cytochrome P450 family 2 subfamily B6	55	0.6
74	GC07M081946	CACNA2D1	Calcium voltage-gated channel auxiliary subunit α21δ	53	0.3
75	GC0XM047635	ELK1	ETS transcription factor ELK1	50	0.26

#### Enrichment Analysis and Construction of a Regulatory Network

**FIGURE 4:**
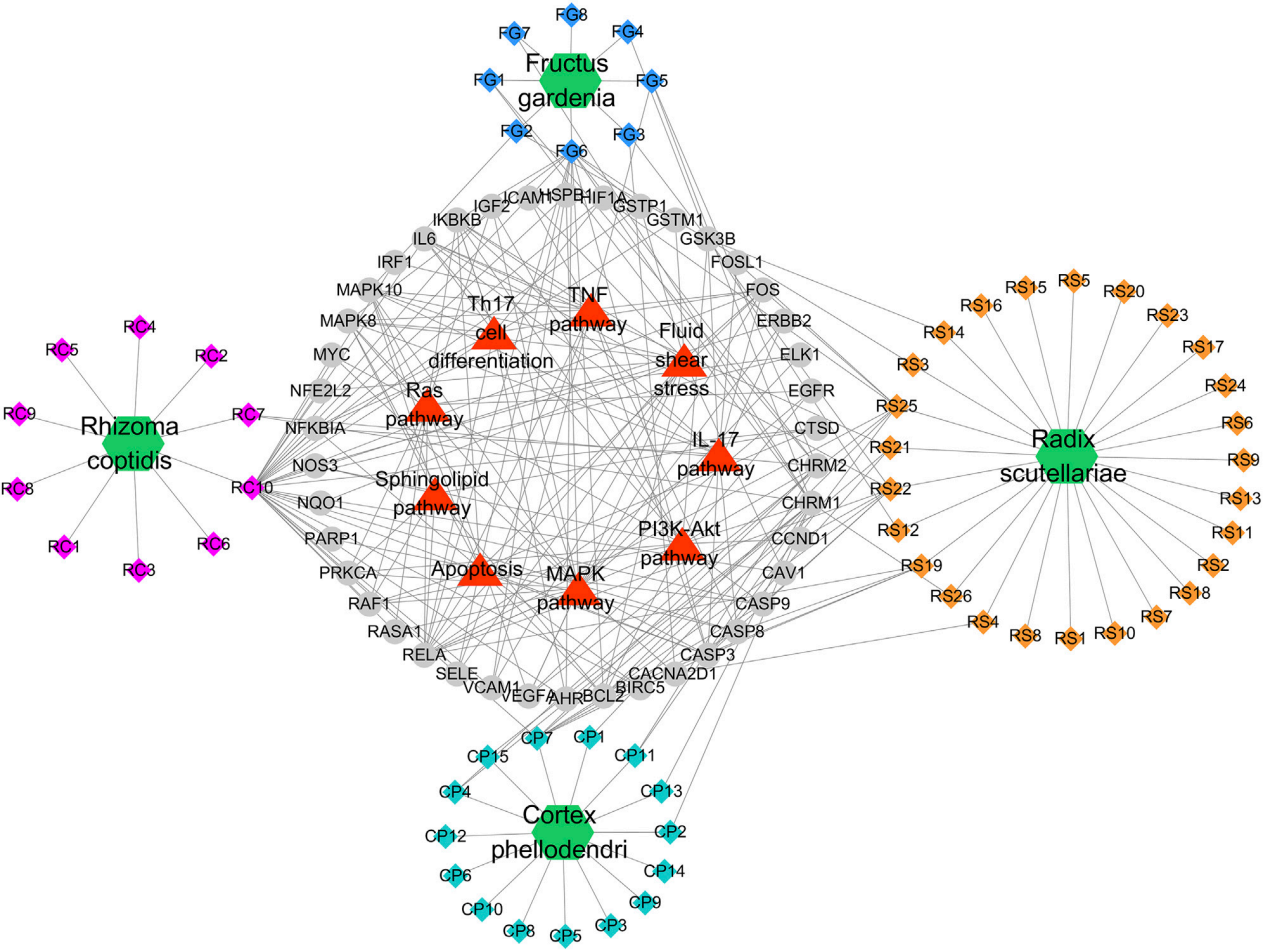
Potential Compound-Targets-Pathway (pC-T-P) network of HLJDT in the treatment of MI. There were 4 kinds of herbs, 59 compounds, 42 predicted targets and 9 signaling pathways on the network. Green hexagon represents 4 kinds of herbs of in HLJDT, every color represents the single medicine. Diamond represents compounds, each color represents one herb in HLJDT; Gray circle represents potential targets, while the red triangle represents the predicted signaling pathways. RC, *Rhizoma coptidis; RS, Radix scutellariae; CP, Cortex phellodendri; FG, Fructus gardenia*.

The potential target genes of HLJDT obtained above were then imported for KEGG pathway enrichment to explore potential signaling pathways for HLJDT in treating MI. The top 20 potential signaling pathways are listed in Figure 4. Among them, nine pathways including the MAPK signaling pathway (hsa04010), the PI3K-Akt signaling pathway (hsa04151), apoptosis (hsa04210), the IL-17 signaling pathway (hsa04657), Th17 cell differentiation (hsa04659), the sphingolipid signaling pathway (hsa04071), the Ras signaling pathway (hsa04014), the TNF signaling pathway (hsa04668), and fluid shear stress (hsa05418) were mainly found to be involved in apoptosis, inflammation, immunity, or oxidative stress biological processes related to MI.

**FIGURE 5:**
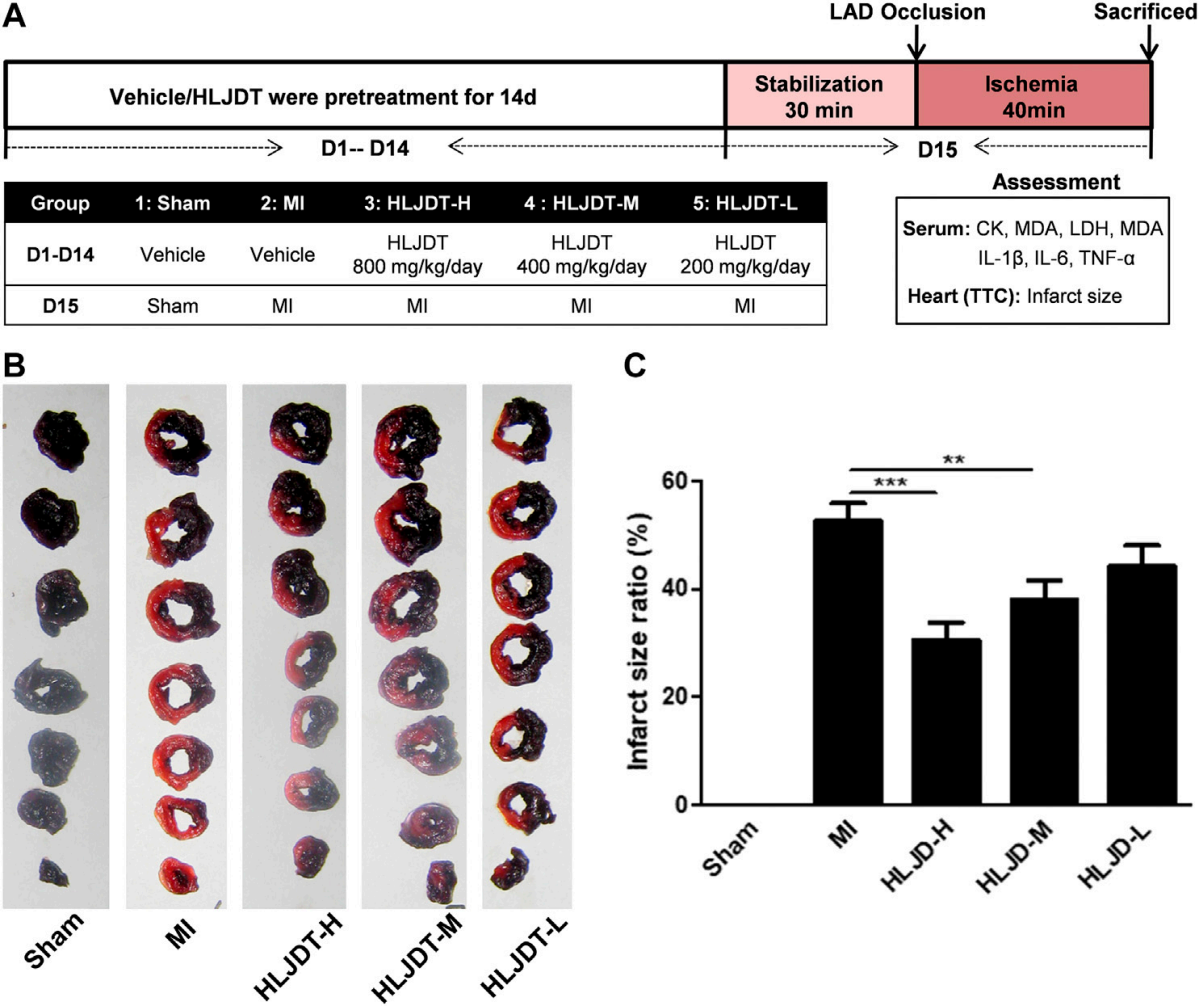
HLJDT reduced infarct size after MI. **(A)**The experimental procedures of *in vivo* MI rat model; **(B)** Representative photographs of TTC stained heart slices obtained after MI; **(C)** Graphic representation of myocardial infarct size. **(B)** Bar graphic representation of statistical analysis myocardial infarct size (n < 6). All data expressed as mean ± SEM, n=6/group. **p<0.01, ***p<0.001 vs. MI group

A global view of the potential compound–target–pathway (C-T-D) network including 114 nodes (4 herbs, 59 compounds, 42 predicted targets, and nine signaling pathways) and 270 edges was constructed (Figure 5) to further clarify the specific mechanism of HLJDT. This network presented the complex relationship among the active components of HLJDT, the target genes, and the related predicted pathways. The top components with the highest number of connections to target nodes were quercetin (RC10) in the rhizoma of *Coptis chinensis*, kaempferol (FG6) in the fructus of *Gardenia jasminoides* J. Ellis; oroxylin (RS21), wogonin (RS22), and baicalein (RS25) in the radix of *Scutellaria baicalensis* Georgi, and β-sitosterol (CP7) in the cortex of *Phellodendron chinense* C.K. Schneid. Meanwhile, some of the target genes were involved in diverse pathways, for example, MAPK8 participated in diverse pathways, including apoptosis, the PI3K-Akt pathway, the TNF pathway, and the MAPK pathway, while IL-17 was involved in the IL-17 signaling pathway, Th17 cell differentiation, and apoptosis, etc. These results indicated that HLJDT probably acted on MI in a multi-target, multi-pathway, and overall integrative mode.

### *In vivo* Validation of the Efficacy of Huang-Lian-Jie-Du-Tang

The rat MI model was established to evaluate the potential myocardial protection of HLJDT. Representative photographs of TTC-stained heart slices and the corresponding quantitative statistical analysis of myocardial infarct size obtained after MI are shown in Figures 2B,C, respectively. The myocardial infarction area of the MI group was significantly increased after ischemia-induced injury compared with the sham group. As expected, the average areas of infarct were significantly reduced after pretreatment with HLJDT in a dose dependent manner.

**FIGURE 6:**
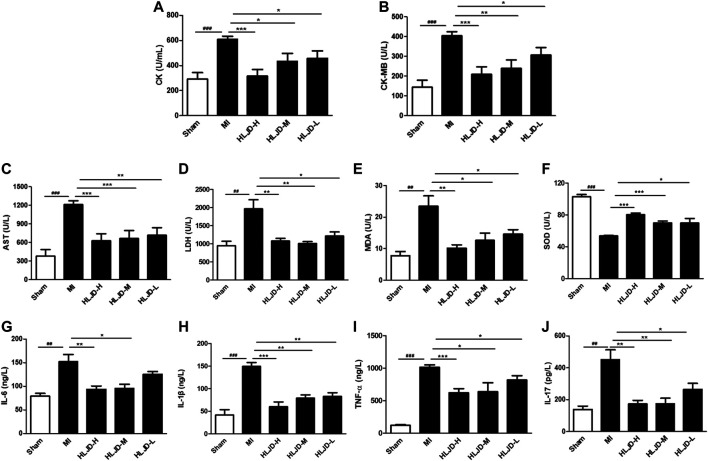
The effect of HLJDT on the release of serum biochemical indicators. **(A)** Creatine kinase (CK); **(B)** CK-MB; **(C)** aspartate aminotransferase (AST); **(D)** lactate dehydrogenase (LDH); **(E)** malondialdehyde (MDA); **(F)** superoxide dismutase (SOD); **(G)** IL-6; **(H)** IL-1β; **(I)** TNF-α; and **(J)** IL-17 (n = 6). Results were presented as mean ± SEM. ##*p* < 0.01, ###*p* < 0.001 vs. Sham group; **p < 0.01, ***p < 0.001 vs. MI group.

Meanwhile, the MI group exhibited elevated levels of cardiac markers such as creatine kinase (CK), CM-KB, aspartate aminotransferase (AST), lactate dehydrogenase (LDH), anti-oxidant enzymes superoxide dismutase (SOD), inflammation, and immune related factors: interleukin-6 (IL-6), IL-1β, IL-17, and TNF-α, while the MI group exhibited reduced levels of malondialdehyde (MDA). Oral administration of HLJDT could significantly improve the above related levels (Figures 6A–H). The results showed that HLJDT exerted a potential preventive effect against MI.

### Metabonomic Analysis

#### Identification of Potential Metabolites

**FIGURE 7:**
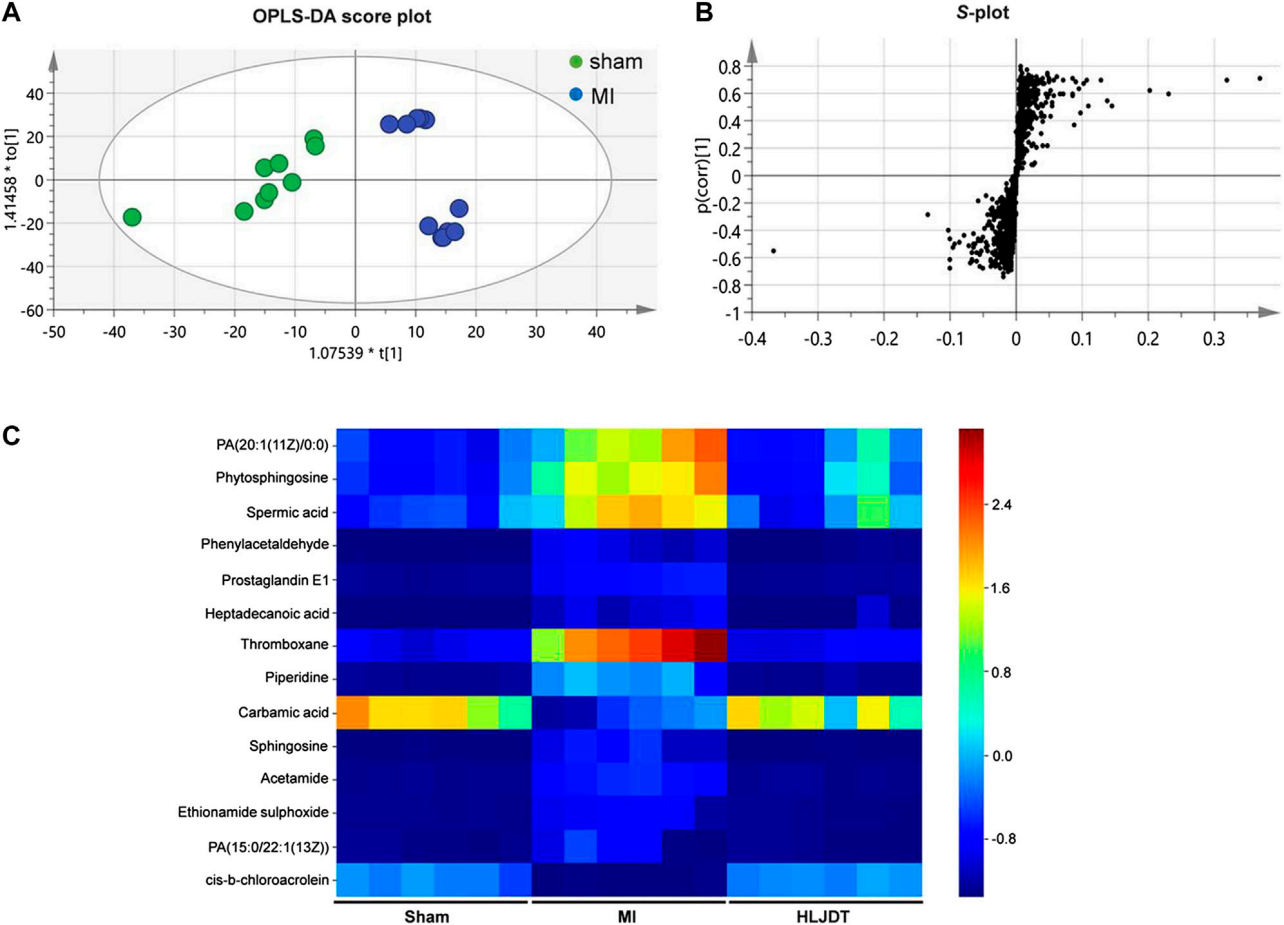
Metabolic data analysis of serum by UPLC-Q-TOF/MS **(A)** OPLS-DA score plot of serum samples from sham and MI model rats in positive mode **(B)** S-plot from OPLS-DA of serum samples from sham and MI model rats in positive mode **(C)** Comparison of normalized mass spectrometric peak intensities for potential biomarkers in different groups. Potential biomarkers were determined by the condition of fold changes larger than 1.5, *p* values less than 0.05, and VIP values larger than 2.0. They were PA (20:1 (11Z)/0:0), phytosphingosine, spermic acid 2, phenylacetaldehyde, prostaglandin E1, heptadecanoic acid, thromboxane, piperidine, carbamic acid, sphingosine, acetamide, ethionamide sulphoxide, PA (15:0/22:1 (13Z)), and *cis*-3-Chloroallyl aldehyde.

To identify the potential metabolic markers of HLJDT, the total ion chromatograms (TICs) of serum were extracted from the sham and MI groups. The OPLS score plot proved that the sham group were significantly different from the MI group (R2X = 0.168, Figure 7A). Moreover, the relative S-plot (Figure 7B) reflected the influences of variables on inter-group difference. Those points far from the origin contribute to inter-group difference significantly and correspond to larger VIP values. The S-plot showed the distribution of biomarkers with significant difference. The endogenous metabolites were identified by comparing them with standard references and mass assignments in online databases (Xu et al., 2015). Potential biomarkers were determined by the condition of fold changes larger than 1.5, *p* values less than 0.05, and VIP values larger than 2.0. They were PA (20:1 (11Z)/0:0), phytosphingosine, spermic acid 2, phenylacetaldehyde, prostaglandin E1, heptadecanoic acid, thromboxane, piperidine, carbamic acid, sphingosine, acetamide, ethionamide sulphoxide, PA (15:0/22:1 (13Z)), and cis-3-Chloroallyl aldehyde. The distribution of 14 metabolites were visually displayed by heatmap (Figure 7C). The corresponding detailed information including chemical name, m/z, formula, and differences are listed in Table 3. These metabonomic results demonstrate the efficacy of HLJDT.

**TABLE 3:** Metabolites identified as potential biomarkers.

No.	m/z	HMDB ID.	Chemical name	Formula	CON vs. MI		HLJDT-H vs. MI		HLJDT-M vs. MI		HLJDT-L vs. MI	
					FC^a^	P^b^	FC	P	FC	P	FC	P
1	464.3132	0062305	PA (20:1 (11Z)/0:0)	C_23_H_45_O_7_P	0.42	**	0.48	*	0.61		0.57	
2	303.2304	0007015	Phytosphingosine	C_18_H_39_O_3_	0.31	*	0.41	*	0.38	*	0.55	
3	482.3192	0013075	Spermic acid 2	C_10_H_20_N_2_O_4_	0.61	*	0.5	**	0.53	**	0.57	*
4	216.9341	0031798	Phenylacetaldehyde	C_8_H_8_O	0.04	*	0.19	*	0.19		0.14	*
5	120.0796	0006236	Prostaglandin E1	C_20_H_34_O_5_	0.34	**	0.3	**	0.25	**	0.32	**
6	288.2896	0002259	Heptadecanoic acid	C_17_H_34_O_2_	0	*	0.23	*	0.17	*	0.47	
7	166.0839	0004827	Thromboxane	C_20_H_40_O	0.32	**	0.26	**	0.24	**	0.21	**
8	86.0966	0034301	Piperidine	C_5_H_11_N	0.2	*	0.15	**	0.1	**	0.11	**
9	140.0662	0003551	Carbamic acid	CH_3_NO_2_	2.11	**	1.72		1.64		2.07	*
10	153.0654	0094673	Sphingosine	C_18_H_37_NO_2_	0.07	*	0.07	**	0.06	*	0.1	*
11	123.0531	0031645	Acetamide	C_2_H_5_NO	0.24	**	0.21	**	0.21	**	0.16	**
12	182.0741	0060624	Ethionamide sulphoxide	C_8_H_10_N_2_OS	0.28	*	0.26	**	0.24	**	0.21	**
13	400.3001	0114,825	PA (15:0/22:1 (13z))	C_40_H_77_O_8_P	0.26	*	0.15	*	0.17	*	0.18	*
14	128.9513	0060458	*cis*-b-chloroacrolein	C_3_H_3_ClO	2.32	**	2.42	*	2.15	**	2.63	*

^a^ Colors were coded according to the values of fold change (FC). Color bar.

^b^ *p*-values: *p < 0.05, **p < 0.01.

#### Pathway Analysis

**FIGURE 8:**
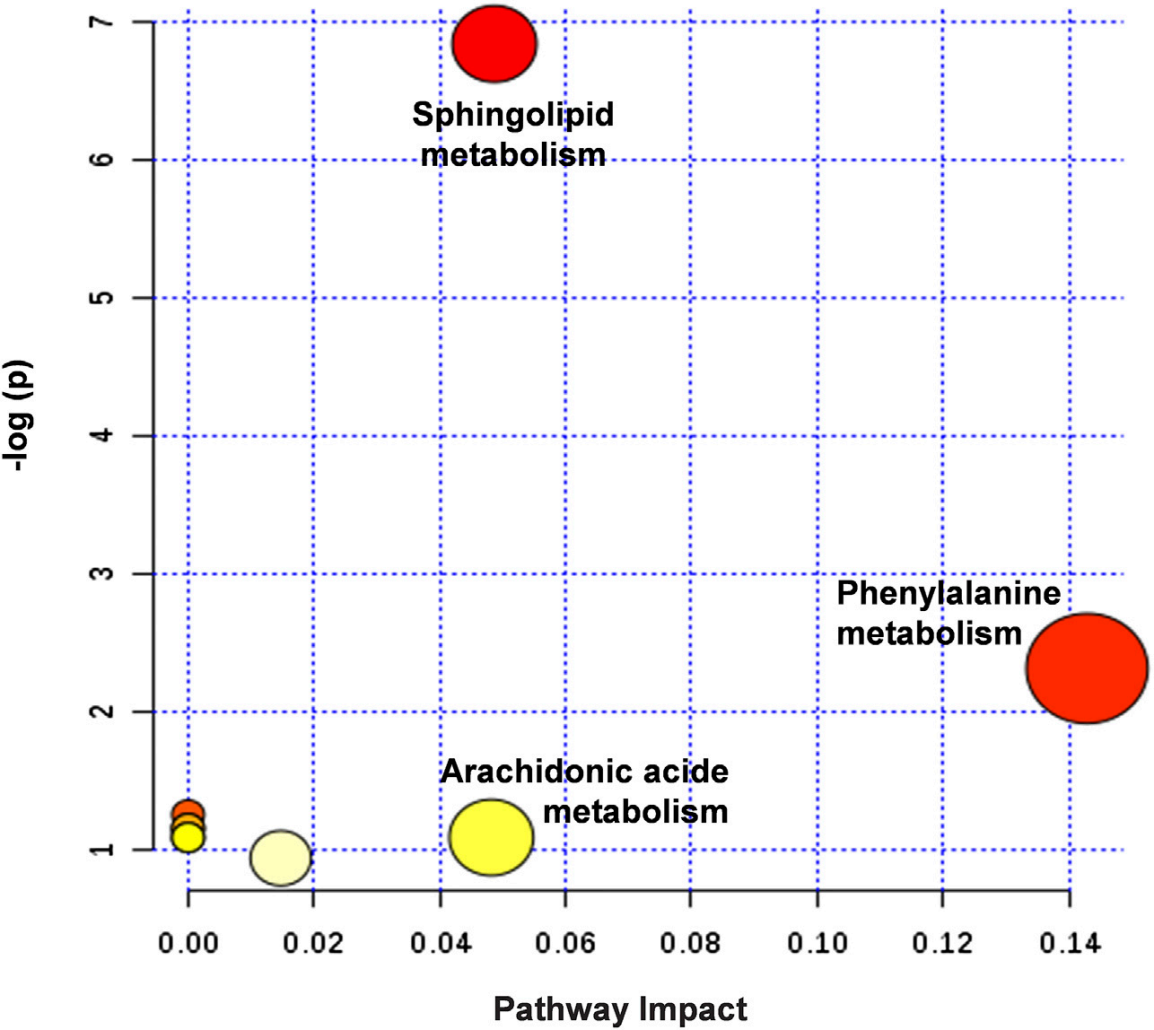
Summary of potential metabonomic pathways in MI injury treated by HLJDT. Among these metabolic pathways, phenylalanine metabolism, sphingolipid metabolism, and arachidonic acid metabolism were filtered out, which were considered as the most significant metabolic pathways.

The metabolic pathways closely related to the 14 difference markers were further analyzed by MetaboAnalyst 4.0. As shown in Figure 8, the top three pathways including phenylalanine metabolism, sphingolipid metabolism, and arachidonic acid metabolism played an important role in regulating MI.

### Integration of Network Pharmacology and Metabonomics

**FIGURE 9:**
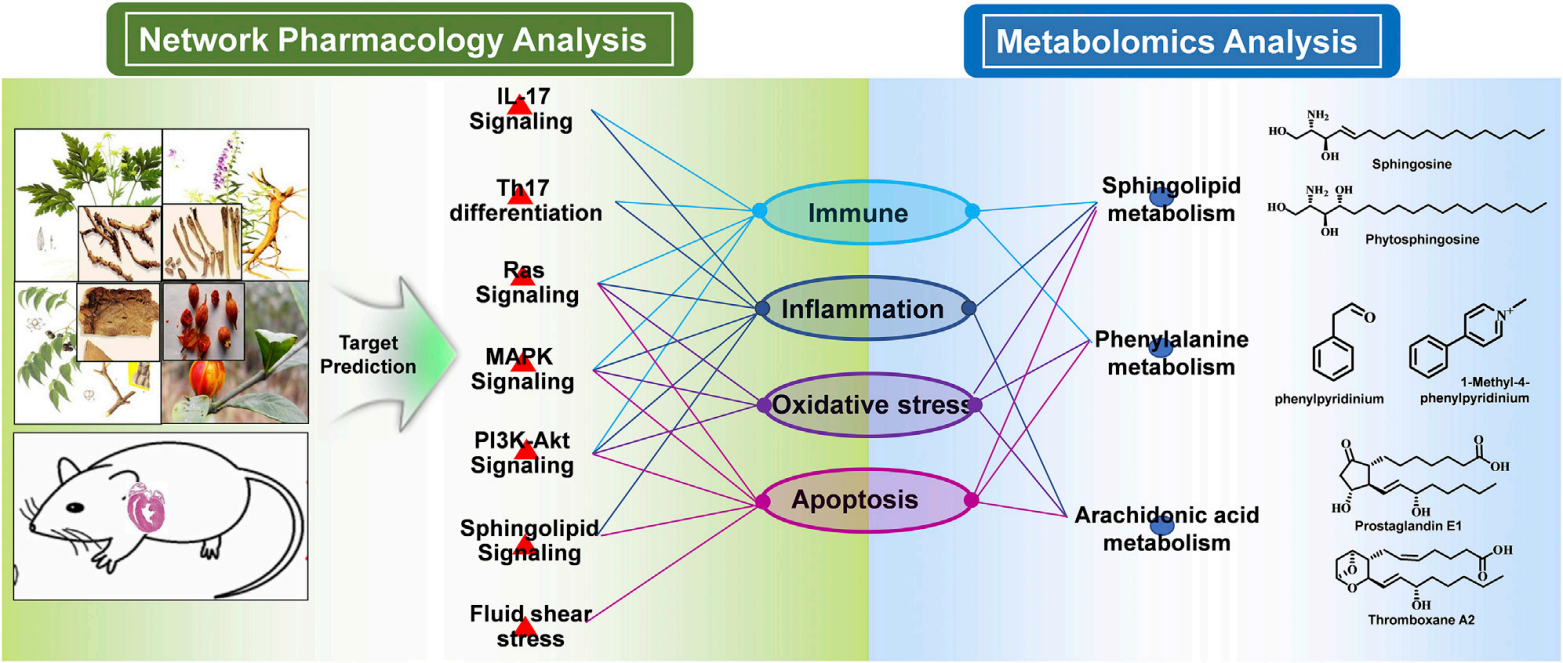
Integrated mechanism of network pharmacology and metabolomic analysis. The main signaling pathways, differentially regulated metabolites, and metabolic pathways were combined logically through the biological functions. From the pathway perspective, the HLJDT targets were significantly enriched in seven pathways including Th17 differentiation, IL-17, RAS, MAPK, Pi3k-Akt, sphingolipid, and fluid shear stress signaling pathways, which were critical in the regulation of the immune, inflammation, oxidative stress, and apoptosis process associated with MI. From the metabolomics perspective, the GO and KEGG pathway enrichment analysis of the differential metabolites revealed consistent results. In summary, network pharmacology analysis suggested that HLJDT could act as an immunomodulatory, anti-inflammatory, antioxidant stress, and anti-apoptotic agent to exert anti-MI effects.

To integrate the above network pharmacology results and metabonomics data and explore the understanding of the underlying mechanisms from a systematic perspective, we focused on the correlation of the signaling pathway and metabolites through their common biological functions related to the pathophysiology of MI. A network with interactions among HLJDT compounds, targets, signaling pathways, metabolic pathways, and biological processes affected by HLJDT on MI is illustrated in Figure 9. The network suggested that the mechanism of HLJDT acting on MI was based on the attenuation of apoptosis, oxidative stress, and immune and inflammatory responses. This could lay the foundation for the biological connotation of the efficacy of the medicine and provide support for the next step of the “disease-syndrome-formula” of HLJDT.

## Discussion

As TCM has the characteristics of a multi-component, multi-target, and multi-pathway synergistic action pattern, it is inappropriate to use the mode of “one drug-one target” to elaborate its mechanism. Network pharmacology is a new strategy for exploring the relationship between drugs and diseases from a global perspective by integrating system biology, multi-directional pharmacology, and computational biology. It is especially suitable for interpreting the complex relationship between drugs, targets, pathways, and diseases (Hopkins, 2008; Li and Zhang, 2013).

In our study, a global view of the potential compound–target–pathway network based on network pharmacology was constructed to investigate the molecular mechanism and potential targets of HLJDT in the treatment of MI. Active ingredients related to HLJDT were obtained from TCMSP (Ru et al., 2014). Protein targets directly affected by myocardial ischemia in HLJDT were screened through the Mendelian genetic database and biological pathway enrichment on the KEGG database (Li et al., 2012). Except for effective ingredients and target genes, the network revealed several meaningful signaling pathways including apoptosis, the PI3K-Akt signaling pathway, the TNF signaling pathway, the MAPK signaling pathway, the IL-17 signaling pathway, Th17 cell differentiation, and the sphingolipid pathway. Biological function enrichment and literature research inferred that these pathways are mainly related to the oxidative stress, apoptosis, inflammation, and immune response involved in MI progression (Whelan et al., 2010; Li et al., 2011; Li et al., 2012; Sinning et al., 2017; Yamamoto et al., 2017). These data provided a preliminary understanding and provoked our interest for further study.

After preliminary identification of the multi-dimensional regulatory network in the treatment of MI, the *in vivo* potency of HLJDT was validated in the MI rat model. HLJDT pretreatment significantly reduced the area of MI, and decreased the ameliorated myocardial injury biochemical markers CK, MDA, and LDH, anti-oxidant enzymes SOD, and inflammation and immune factors: IL-6, IL-1β, IL-17, and TNF-α. These results suggested that HLJDT exerted a potential preventive effect against MI.

Metabonomics is a discipline in which the metabolic changes of organisms are quantitatively detected at different times and in multiple directions under the conditions of pathophysiological stimulation and genetic factors change, and the mechanism of gene function regulation is explored by measuring the metabolic map of the whole organism (Jia et al., 2006). Metabonomics can be used to explore the correlation between metabolites and physiological and pathological changes through high-throughput analysis of metabolites in the body, and clarify the interaction of complex systems and responses to the outside body. Metabonomics mainly focus on the variety and quantity of endogenous small molecule metabolites (generally referred to MW < 1,000) under the action of internal and external factors (such as disease invasion, drug intervention, and environmental change, etc.) and their interrelations. Metabonomics studies the human body as one system, and has unique advantages in revealing the mechanism of complex diseases and drug metabolism mode, thus providing new ideas for the study of the complex system of TCM (Shyur and Yang, 2008).

Metabonomic analysis showed that there were 29 metabolites with significant differences between the sham group and MI group, where 14 biomarkers were regulated by HLJDT pretreatment, namely (PA (20:1 (11Z)/0:0), phytosphingosine, spermic acid 2, phenylacetaldehyde, prostaglandin E1, heptadecanoic acid, thromboxane, piperidine, carbamic acid, sphingosine, acetamide, ethionamide sulphoxide, PA (15:0/22:1 (13Z)), and *cis*-3-Chloroallyl aldehyde. These metabolites were mainly involved in three metabolic pathways including sphingolipid metabolism, phenylalanine metabolism, and arachidonic acid (AA) metabolism (Gross et al., 2005). The existing research shows that sphingolipid is a kind of complex compound in the skeleton and plays an important role in maintaining cell growth, signal transduction, and inflammation (Egom et al., 2012). An abnormal sphingolipid may cause atherosclerosis, cardiomyopathy, cancer, and other diseases (Jiang et al., 2011; Glaser and Fandrey, 2018). Ceramide is at the center of intracellular sphingolipid metabolism. It has the biological function to induce apoptosis, regulating cell differentiation, cellular immunity, and inflammatory response (Bai et al., 2007). The AA metabolic pathway plays an important role in the process of MI, as the main metabolites prostacyclin and thromboxane are closely related to the biological processes of vasodilation, anti-inflammatory, and anti-oxidation during the development of MI (Gross et al., 2005).

Notably, in the main target genes and differentially regulated makers, the sphingolipid signaling pathway was found to be the only shared pathway. According to the literature, sphingolipids, the critical composition of the cell membrane, regulates cellular growth, differentiation, and aging (Mendelson et al., 2014). It includes sphingomyelin, cerebroside, and ganglioside. Among them, sphingomyelin plays a significant role in acute leukemia disease (Li et al., 2014). Some studies have indicated that sphingolipids were involved in inflammation, apoptosis, and cellular immunity response (Testai et al., 2014). Sphingosine 1-phosphate (S1P) is a biologically active sphingolipid and plays an important role in tumor genesis, the cardiovascular system, and the immune system through distinct signal transduction pathways (Mendelson et al., 2014). During the process of MI, S1P may play a significant role in the biological process of vascular endothelial protection, the inhibition of inflammation, and oxidative stress. Here we can reasonably speculate that during the early stage of MI, the inflammatory cells are stimulated by hypoxia to produce many kinds of proinflammatory factors. With the continuous activation and release of inflammatory factors, a positive feedback effect is formed, which leads to the loss of inflammatory response and serious myocardial damage (Knapp et al., 2012). HLJDT was proven to reduce the production of proinflammatory factors including IL-1β, TNF-α, IL-6, as well as IL-17, thus it is of great significance in alleviating ischemia.

## Conclusion

The present study demonstrated the myocardial protection of HLJDT. An integrative approach of network pharmacology and metabonomic was performed to investigate the biological mechanisms of HLJDT for treating MI. By comprehensive analysis of the potential compound–target–pathway network and metabolic pathway enrichment, we speculated that the action mechanisms of HLJDT were attributed to the biological process of oxidative stress, apoptosis, and immune and inflammatory responses by regulating the sphingolipid pathway, PI3K-Akt pathway, and IL-17 signaling pathway. Meanwhile, the integrated network indicated that the top ingredients with the highest number of connections to target nodes were quercetin in the rhizoma of *Coptis chinensis* Franch, kaempferol in the fructus of *Gardenia jasminoides* J. Ellis, oroxylin, wogonin, and baicalein in the radix of *Scutellaria baicalensis* Georgi, and β-sitosterolin in the cortex of *Phellodendron chinense* C.K. Schneid. The results demonstrated the myocardial protection effect of HLJDT on ischemia with a multicomponent-multitarget mode. This study highlights the reliability and effectiveness of a network pharmacology-based approach that identifies and validates the complex of natural compounds in HLJDT for illustrating the mechanism of action in the treatment of MI.

## Data Availability

The raw data supporting the conclusion of this article will be made available by the authors, without undue reservation, to any qualified researcher.
