# Supplementary figures and images for: Integrated Network Pharmacology and Metabonomics to Reveal the Myocardial Protection Effect of Huang-Lian-Jie-Du-Tang on Myocardial Ischemia
Source: Front Pharmacol. 2021 Feb 4;11:589175. doi: 10.3389/fphar.2020.589175 (PMC7890363; doi:10.3389/fphar.2020.589175)

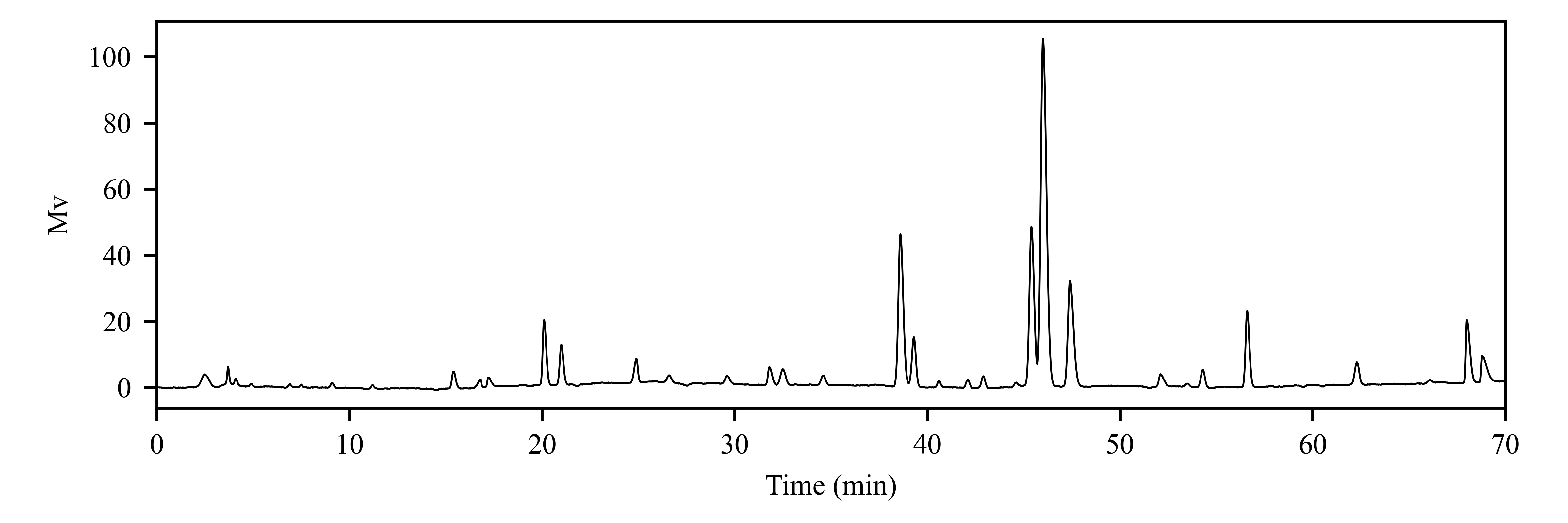

Supplement: Supplementary file 1 [file Image1_v1.JPEG]
